# Divergent genes encoding the putative receptors for growth hormone and prolactin in sea lamprey display distinct patterns of expression

**DOI:** 10.1038/s41598-020-58344-5

**Published:** 2020-02-03

**Authors:** Ningping Gong, Diogo Ferreira-Martins, Stephen D. McCormick, Mark A. Sheridan

**Affiliations:** 1 Department of Biological Sciences, Texas Tech University, Lubbock, TX 79409 USA; 2 Department of Biology, University of Massachusetts, Amherst, MA USA; 3 U.S. Geological Survey, Leetown Science Center, S.O. Conte Anadromous Fish Research Laboratory, Turners Falls, MA 01376 USA

**Keywords:** Data mining, Molecular evolution, Animal physiology

## Abstract

Growth hormone receptor (GHR) and prolactin receptor (PRLR) in jawed vertebrates were thought to arise after the divergence of gnathostomes from a basal vertebrate. In this study we have identified two genes encoding putative GHR and PRLR in sea lamprey (*Petromyzon marinus*) and Arctic lamprey (*Lethenteron camtschaticum*), extant members of one of the oldest vertebrate groups, agnathans. Phylogenetic analysis revealed that lamprey GHR and PRLR cluster at the base of gnathostome GHR and PRLR clades, respectively. This indicates that distinct GHR and PRLR arose prior to the emergence of the lamprey branch of agnathans. In the sea lamprey, GHR and PRLR displayed a differential but overlapping pattern of expression; GHR had high expression in liver and heart tissues, whereas PRLR was expressed highly in the brain and moderately in osmoregulatory tissues. Branchial PRLR mRNA levels were significantly elevated by stage 5 of metamorphosis and remained elevated through stage 7, whereas levels of GHR mRNA were only elevated in the final stage (7). Branchial expression of GHR increased following seawater (SW) exposure of juveniles, but expression of PRLR was not significantly altered. The results indicate that GHR and PRLR may both participate in metamorphosis and that GHR may mediate SW acclimation.

## Introduction

The receptors for growth hormone (GHR) and prolactin (PRLR) belong to the single-chain subgroup of the superfamily of class-I cytokine receptors^1,2^ and are classified based on their preferential ligand binding and phylogenetic analyses^3^. A single gene encoding a putative GHR or PRLR (GHR/PRLR) was characterized from lamprey^4,5^, one of the extant agnathan lineages (including lamprey and hagfish) that diverged from the lineage leading to gnathostomes. Based on phylogenetic analysis, the GHR/PRLR was proposed as the common ancestor of GHR and PRLR or as a member of either GHR or PRLR clades^4–6^. GHR and PRLR were clearly present after the divergence of gnathostomes from a basal vertebrate and have gone through a series of gene duplication events^6,7^. The basal teleost tetraploidization gave rise to 2 subtypes of GHR, while 3 or 4 subtypes of GHR and 2 subtypes of PRLR arose in the salmonid fish from the salmonid tetraploidization^4,6^. A class-I cytokine receptor with unknown ligand, named tentatively as cytokine receptor family member A4 (CRFA4)^8^, has relatively high sequence identity and similar gene structure to GHR and PRLR, and thus the CRFA4 sequences cluster with the gnathostome GHR clade in the phylogeny from a previous study^6^, indicating evolutionary relationship with GHR.

GHR signalling has well-recognized impacts on regulation of growth and metabolism, whereas PRLR has a wide range of actions including effects on osmoregulation, parental behaviours, reproduction, metabolism, and immune responses^9,10^. The GHR and PRLR subtypes are functionally distinct in teleosts; the mRNA levels of GHR subtypes are regulated differently by fasting^11^ and the mRNA levels of PRLR subtypes differentially vary after salinity changes^12^. Both GHR and PRLR have been shown to be involved in osmoregulation (seawater and freshwater acclimation, respectively) in teleost fishes by regulating cell proliferation and differentiation of ionocytes and other osmoregulatory cells^13^ and by regulating the abundance of ion transporters and other osmoregulatory factors^14,15^.

Sea lamprey (*Petromyzon marinus*) have an anadromous life cycle that involves fresh water (FW) and seawater (SW) phases. Filter-feeding ammocoetes (lamprey larvae) hatch and live in the soft sediment of FW streams, generally for 3–7 years. The ammocoetes then undergo a radical metamorphosis composed of seven stages, involving the reorganization of organs and the remodelling of body to enable a parasitic feeding phase in the ocean^16,17^. Increased salinity tolerance occurs during metamorphosis following the development of SW-type ionocytes with high levels of Na^+^/K^+^-ATPase^18,19^. It is not known whether the previously identified lamprey GHR/PRLR^4,5^ protein plays a role in the acquisition of salinity tolerance during metamorphosis and the subsequent SW exposure.

The growth hormone (GH) gene has been sequenced in the sea lamprey, and the GH protein purified from the sea lamprey pituitary gland has been shown to stimulate insulin-like growth factor (IGF) in the sea lamprey liver^20^. This function thus appears to be conserved in both agnathans and gnathostomes. To date, there is no information about prolactin (PRL) in the annotation of genome assemblies from the sea lamprey^21^, Arctic lamprey (*Lethenteron camtschaticum*)^5,22^ and inshore hagfish (*Eptatretus burgeri*)^23^, while a PRL-releasing peptide has been detected in the brain of inshore hagfish^24^. However, it was concluded that the early expansion of the GH family–including GH, an ancestral PRL, and somatolactin (SL)–occurred before the divergence of the chondrichthyan and osteichthyan lineages^5,25^, or even earlier before the vertebrate radiation^26^. There may be asynchrony in hormone and receptor gene duplication that occurred during vertebrate evolution^26^.

As only an extracellular GHR/PRLR domain had been identified in the sea lamprey^4^, our aim was to complete its full-length sequence and to include the transmembrane and intracellular sequences for a more complete phylogenetic analysis. In the process, we found that there are two distinct genes in the GHR/PRLR family in the sea lamprey. We further determined the mRNA levels of the receptor genes among tissues, as well as the patterns of expression of the mRNAs during metamorphosis and following SW exposure.

## Results

### Gene sequences of the GHRs and PRLRs from lamprey and hagfish

The ORFs of putative GHR and PRLR from sea lamprey include 2208 and 1890 nucleotides, encoding 736 and 629 amino acids, respectively. The sea lamprey putative PRLR gene (GenBank no. MF685336) is located on Pmar_germline 1.0 scaffold 00023 (GenBank no. PIZI01000023.1) and has 96% sequence identity with the ortholog from Arctic lamprey located on LetJap1.0 scaffold 000254 (GenBank no. KE993925.1). The extracellular domain of the sea lamprey putative PRLR is identical to the previously identified sea lamprey GHR/PRLR protein^4^.

The sea lamprey putative GHR (GenBank no. MK593139) is located on Pmar_germline 1.0 scaffold 00043 (GenBank no. PIZI01000043.1) and has 25% sequence similarity with the lamprey PRLR. The GHR ortholog from Arctic lamprey is located on LetJap1.0 scaffold00568 (GenBank no. KE994239.1). The predicted gene is incomplete and lacks the sequences encoding the characteristic WSXWS motif and the transmembrane domain, due to the gaps in the genome assembly of Arctic lamprey. The two lamprey GHR orthologs share around 90% identity in amino acid composition. The lamprey GHR and PRLR share between 25% and 30% amino acid identity with gnathostome GHRs and PRLRs.

A partial sequence of a GHR/PRLR-like gene from inshore hagfish was found in the Ensembl genome browser (Ensembl no. ENSEBUG00000011491). Through searching the inshore hagfish genome assembly^23^, we obtained two predicted exons likely encoding the intracellular region of the hagfish GHR/PRLR-like. The predicted sequences in the extracellular and intracellular parts of the hagfish GHR/PRLR-like have slightly higher identities to the lamprey GHR sequence (30% and 26%, respectively) than to the lamprey PRLR (25% and 20%, respectively). The sequences of GHRs and PRLRs from the lampreys and hagfish GHR/PRLR-like are aligned in Supplemental Fig. S1.

### Phylogenetic analysis of GHR and PRLR genes

**Figure 1:**
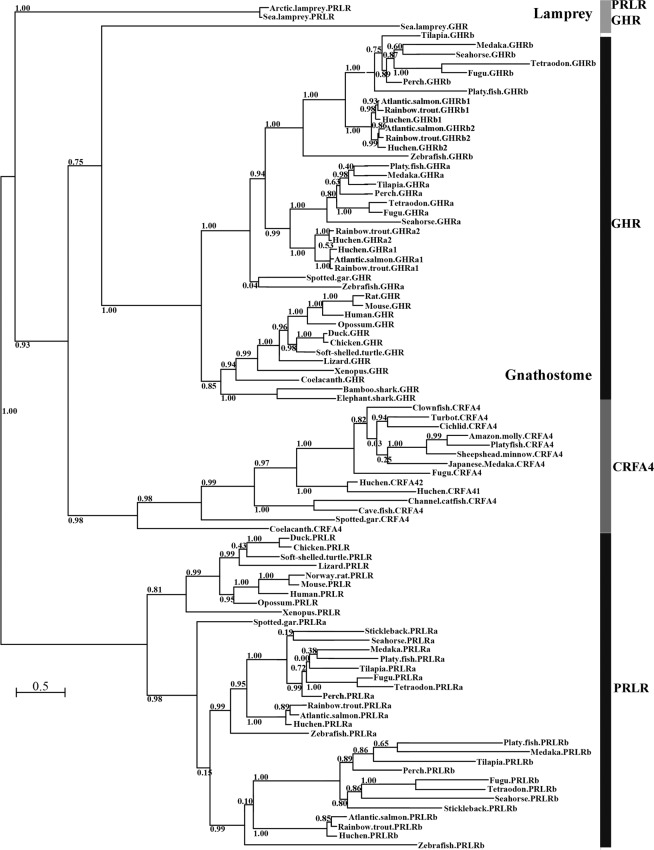
Phylogenetic maximum likelihood tree of the lamprey putative GHR and PRLR as well as selected gnathostome GHRs, PRLRs and CRFA4s. The tree is supported by aLRT with SH-like node supports. Nodes with support values ≤ 0.5 were considered uninformative. The branch lengths of the tree are proportional to the number of substitutions per site. The tree is displayed as a midpoint-rooted phylogram. A complete species list and transcript accession numbers in GenBank and Ensembl are listed in Supplemental Table S2.

The phylogenetic analysis including the lamprey receptors and the sequences of GHR, PRLR and CRFA4 from gnathostomes shows four main branches at the basal node, the clades of GHR, PRLR and CRFA4 from gnathostomes and the branch of lamprey PRLRs, while the sea lamprey GHR clusters at the base of gnathostome GHR clade with weak support (0.75; Fig. 1). The partial sequences of the GHR ortholog from Arctic lamprey and the GHR/PRLR-like from inshore hagfish were excluded from the phylogenetic analysis, as the inclusion of those partial sequences created extensive gaps in the sequence alignment and reduced the approximate Likelihood Ratio Tests (aLRT) supports.

### Exon-intron organization of sea lamprey GHR and PRLR genes and conserved sequence motifs

**Figure 2:**
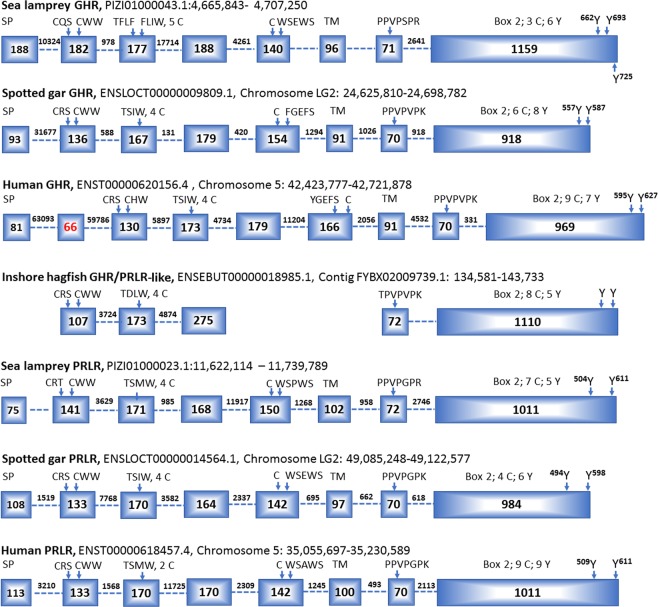
Exon-intron organization of the GHR and PRLR genes from sea lamprey, spotted gar and human as well as from the partial inshore hagfish GHR/PRLR-like sequence (missing the sequences in exons 1, 5 and 6). The human GHR gene has an additional exon (in red). The accession numbers in GenBank or Ensembl and genomic/chromosome loci are listed. Box indicates exon in base pairs; intron length is indicated between two boxes, and some are missing because of the gaps in genome assemblies (including introns 5–6 and 6–7 in sea lamprey GHR, intron 7–8 of the hagfish GHR/PRLR-like partial gene, and intron 1–2 of the sea lamprey PRLR); arrows approximately indicate the motif sites; SP: signalling peptide; TM: transmembrane domain; other capital letters are single-letter codes of amino acids. The number indicating the position of the tyrosine residue is based on the first amino acid of the ORF with the signal peptide.

The GHR and PRLR genes from sea lamprey have similar exon-intron organization (Fig. 2), each with eight exons in the ORF. This organization is also found in gnathostome GHRs and PRLRs as exemplified by human (*Homo sapiens*) and spotted gar (*Lepisosteus oculatus*) GHRs and PRLRs (Fig. 2). Note that primate GHR genes have an additional intron in 5′. The 1^st^ exon encodes the signal peptide (SP). The following three exons encode the putative ligand binding domain, including immunoglobin-like (Ig-like) and fibronectin type 3 (FN3) regions. The 5^th^ exon encodes the characteristic “WSXWS” motif, and the following exon encodes a predicted transmembrane (TM) domain. The last two exons encode the intracellular domain and the 3′ untranslated region (Fig. 2).

Several conserved motifs are revealed through amino acid alignment of the lamprey PRLR and GHR with other vertebrate receptors (Fig. 2 and Supplemental Figs. S1–S3). These include the CWW, T(S/A)XW and WSXWS motifs in the extracellular domain, the so-called “box 1” motif [PPVPXP(K/R)] in the intracellular domain, and several Tyr residues in the C-terminal intracellular domain (Fig. 2). The WSXWS motif appears in lamprey GHR and PRLR, similar to gnathostome PRLRs and CRFA4s; this motif is substituted by (F/Y)G(E/D)FS in the gnathostome GHRs (Fig. 2 and Supplemental Fig. S3). Similarly, the hagfish GHR/PRLR-like gene contains 3 exons encoding the Ig-like and FN3 domains, including the CWW and TDLW motifs, as well as 2 exons encoding the intracellular part including the box 1 (Fig. 2).

Sea lamprey GHR and PRLR have 11 and 8 Cys residues in their extracellular domains, respectively. Six of these Cys residues in the lamprey GHR and PRLR as well as in hagfish GHR/PRLR-like are aligned with the Cys residues in the ligand binding domain (LBD) of spotted gar GHR and human GHR (Fig. 2 and Supplemental Figs. S1 and S3). Moreover, two C-terminal Tyr residues in the intracellular domain are conserved for GHR and PRLR across both agnathans and gnathostomes (Fig. 2 and Supplemental Figs. S1–S3).

### Tissue distribution of sea lamprey GHR and PRLR mRNAs

**Figure 3:**
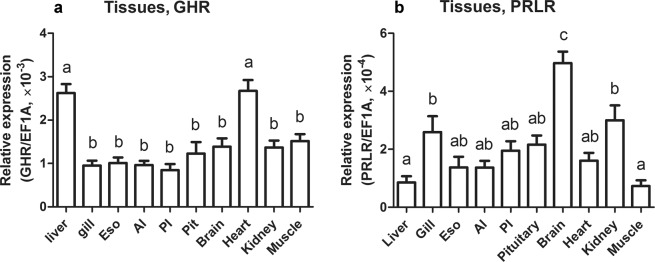
Distribution of lamprey GHR (**a**) and PRLR (**b**) mRNAs among selected tissues of sea lamprey juveniles. Abbreviations: Eso, esophagus; AI, anterior intestine; PI, posterior intestine; Pit, pituitary gland. GHR and PRLR transcript counts were calculated by relating C_T_ value to standard curves and then normalized to reference genes. Data are presented as mean ± SEM (n = 4–6); groups with different letters are significantly different.

Sea lamprey GHR and PRLR displayed a differential but overlapping pattern of expression among tissues. Expression of sea lamprey GHR mRNA was the highest in liver and heart tissues (Fig. 3a). Levels of sea lamprey PRLR mRNA were most abundant in the brain, followed by moderate levels in the gill and kidney, and the lowest expression in the liver and muscle (Fig. 3b). Notably, steady-state levels of GHR mRNA were generally higher than those of PRLR mRNA (Fig. 3).

### Changes in sea lamprey GHR and PRLR mRNA expression during metamorphosis and seawater acclimation

**Figure 4:**
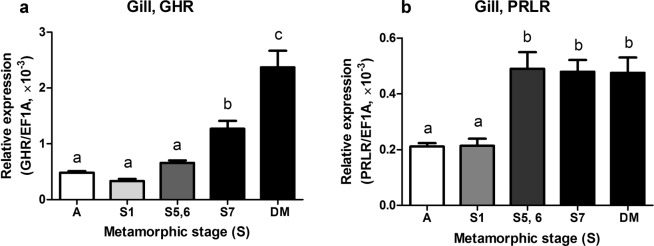
Branchial expression of lamprey GHR (**a**) and PRLR (**b**) mRNAs during the metamorphosis of sea lamprey. Abbreviations and group designations: A, the ammocoetes (larvae; n = 31); S1, S5, S6, S7 and DM groups, the transformers at metamorphic stage 1 (n = 9), 5 (n = 5), 6 (n = 4), 7 (n = 9), and the post-metamorphic downstream migrants (n = 11), respectively. Data are presented as means ± SEM; groups with different letters are significantly different. The P-values of these comparisons are indicated in the Results.

The sea lamprey ammocoetes (larvae) and transformers at various metamorphic stages (1–7) were sampled for gene transcription analysis. During metamorphosis, the GHR mRNA levels in the gill were elevated in the final metamorphic stage (stage 7) compared to ammocoetes and to transformers at stages 1, 5 and 6 (P < 0.0001, Fig. 4a). Branchial GHR mRNA levels were further upregulated (P < 0.0001) in the post-metamorphic downstream migrants approximately 220 km from the ocean. PRLR mRNA levels in the gill were elevated to maximum levels by stage 5 compared to ammocoetes and stage-1 transformers (P < 0.0001) and remained elevated throughout the later stages of metamorphosis and in post-metaphoric downstream migrants (P < 0.0001; Fig. 4b).

**Figure 5:**
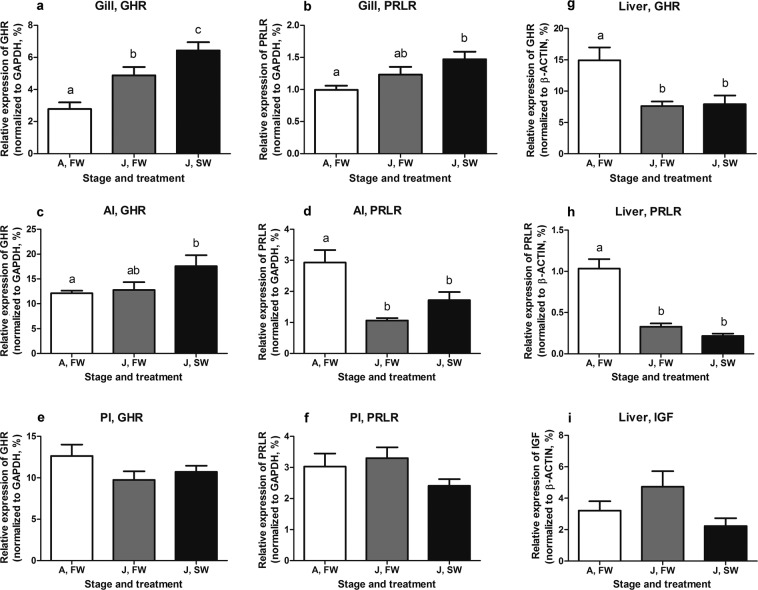
Expression of lamprey GHR and PRLR mRNAs in the gill (**a**,**b**), anterior intestine (**c**,**d**), posterior intestine (**e**,**f**), and the liver (**g**,**h**) as well as of lamprey insulin-like growth factor (IGF) mRNA in the liver (**i**) of freshwater ammocoetes (n = 7) and the juveniles of sea lamprey acclimated to fresh water (n = 8) and seawater (n = 10). Abbreviations: FW, fresh water; SW, seawater; A, ammocoetes; J, juveniles. Data presented as means ± SEM; groups with different letters are significantly different. The P values of these comparisons are indicated in the Results.

Sea lamprey juveniles were acclimated to seawater (SW; salinity 35 ppt) for 3 weeks and sampled for gene transcription analysis. Freshwater (FW) ammocoetes are intolerant to SW and thus not subjected to the SW acclimation^19^. In this experiment, GHR mRNA levels in the gill increased following exposure to SW (P = 0.048, Fig. 5a); whereas, the levels of PRLR mRNA did not change significantly following SW exposure (Fig. 5b). Moreover, the mRNA levels of branchial GHR were upregulated in juveniles compared to ammocoetes (P = 0.0001, Fig. 5a).

In the anterior intestine (AI), GHR mRNA levels were upregulated in SW juveniles compared to ammocoetes (P = 0.04, Fig. 5c), whereas, levels of PRLR mRNA were decreased in juveniles compared to ammocoetes (P = 0.0002, Fig. 5d). In the posterior intestine (PI), neither GHR nor PRLR mRNAs levels were different between ammocoetes or FW juveniles or between FW and SW juveniles (P = 0.17, Fig. 5e; P = 0.15, Fig. 5f).

Hepatic GHR and PRLR mRNA levels were both downregulated in juveniles compared to ammocoetes (P = 0.006, Fig. 5g; P < 0.0001, Fig. 5h), but there was no effect of SW exposure on hepatic expression of GHR or PRLR mRNAs. Hepatic IGF mRNA levels did not vary significantly among the three groups (P = 0.088, Fig. 5i).

## Discussion

Through extensive screening of lamprey genome assemblies, we identified two genes encoding single-chain class-I cytokine receptors (sharing ~25% sequence identity) from sea lamprey and Arctic lamprey; one novel gene and one containing a sequence identical to the previously reported sea lamprey GHR/PRLR extracellular domain^4^. Our phylogenetic analysis indicates that the novel gene appears to be orthologous to gnathostome GHR, and thus we designated it as putative GHR (We use the term putative until ligand binding has been characterized.). There is no definitive information from the phylogeny about the evolutionary relationship between the gene containing the sequence previously referred to as GHR/PRLR and the gnathostome PRLR and CFRA4. A pattern of basal divergences and exclusive clustering of lamprey sequences similar to that observed in this study has been previously observed with other lamprey genes, such as Hox, ParaHox and KCNA, and sometimes phylogenetic analysis was not informative^21,22,27,28^. The CRFA4 sequences are generally shorter than GHR and PRLR sequences and particularly have shorter intracellular domains; for example, the intracellular domain of Japanese medaka CRFA4 has 165 and 115 amino acids fewer than in that of the two sea lamprey receptors. Thus, the lamprey receptor previously referred to as GHR/PRLR is designated as putative PRLR. We recognize that our phylogenetic analysis may have artefacts caused by the low sequence conservation (around 25% identity between the lamprey and gnathostome receptors) and peculiar amino acid composition, due to codon usage bias tending to have higher frequencies for guanine-cytosine (GC)-rich codons, as noted by others^27,28^. Given these limitations, it is also possible that the lamprey PRLR may be a different subtype in the family that has been lost from the jawed vertebrates. Nevertheless, the identification of the two distinct genes from the lampreys indicates that the divergence of GHR and PRLR occurred prior to emergence of the gnathostome lineages from an ancestral vertebrate leading to lamprey. The mechanism and timing of this split is unknown. It is possible that the divergence occurred prior to the emergence of a common agnathan ancestor (prior to or during the 1 R/2 R duplication) or after lamprey diverged from a common agnathan ancestor.

The lamprey GHR clusters with the gnathostome GHR clade, indicating that they are evolutionarily related. The sea lamprey GHR has the characteristic WSXWS motif; however, the amino acid composition of this motif was substituted by (F/Y)G(E/D)FS in the gnathostome GHR. It seems that this motif evolved after the agnathan-gnathostome split. The CRFA4 sequences clustering with the GHR clade was reported in a previous study^6^; however, our phylogenetic tree adding lamprey PRLR and GHR sequences did not show that the CRFA4 clade clusters closer to the GHR clade than to the PRLR clade. Despite exhaustive searching, we could not identify a sequence in the lamprey genome that clustered within the CRFA4 clade. CRFA4 may be an ancestral family member but lost in the lamprey^6^ or arose after the divergence of lamprey lineage from a common agnathan ancestor. The loss of CRFA4 may have occurred secondarily in some lineages, such as in cartilaginous fish and tetrapods^6^.

The lamprey GHR and PRLR genes are located on different scaffolds in the genome assembly of sea lamprey as well as in the Artic lamprey genome assembly; whereas, the gnathostome GHR and PRLR genes are located on the same chromosome (the distances between the two are 7.37 MB, 2.82 MB and 24.47 MB in human, chicken and spotted gar chromosomes, respectively)^4,6^. We conducted conserved synteny analyses for GHR and PRLR in the genomes of human, spotted gar, lampreys and inshore hagfish, but found that most of the neighboring gene families identified in the synteny analysis of GHR and PRLR from human and spotted gar^4,6^ do not have comparable loci in lamprey genomic scaffolds. The mixed patterns of syntenic genes in the lampreys also have been observed in other studies^21,28,29^, leading some to suggest that synteny may not be useful for informing the orthology of lamprey and gnathostome genes^28^. The slightly higher sequence identity of inshore hagfish GHR/PRLR-like to the lamprey GHR than to the lamprey PRLR together with the identification of three conserved Tyr residues in the hagfish GHR/PRLR-like and the lamprey GHR (Supplemental Figs. S1) indicates that the hagfish protein may be the ortholog of lamprey GHR.

The previously reported GHR/PRLR gene from sea lamprey contains the first four exons encoding the extracellular domain and a following intron sequence to generate a premature stop codon and 3′ UTR^4^. This truncated isoform has the TSXW motif that is part of the LBD for the full-length PRLR^4,6^, but does not have the WSXWS motif that is necessary for protein folding and stability of a cytokine receptor but not directly involved in ligand binding^1,30^. Thus, the truncated isoform is likely expressed as a soluble protein to serve as a ligand-binding protein, as alternatively splicing of pre-mRNA has also been characterized as one of the mechanisms in the generation of soluble hormone-binding protein^31^.

In the LBD, four Cys residues are conserved in the PRLRs of spotted gar, frog, chicken, and humans; whereas, the LBD of GHRs in these groups possess 6 Cys residues, forming three disulfide bonds to produce three short loops^4^. Those 6 residues are all conserved in the lamprey GHR and PRLR as well as in the hagfish GHR/PRLR-like. This indicates that the last 2 Cys residues in the LBD of PRLRs may have been lost during the evolution of jawed vertebrates.

In the intracellular regions, the lamprey GHR and PRLR share low amino acid identity (less than 20%) with gnathostome GHRs and PRLRs, but the box 1, box 2 and C-terminal Tyr residues for signal transduction seem to be well conserved. The proline-rich box 1 [PP(V/I)PXP(R/K)] motif is required for Janus kinase (JAK) association in GHR and PRLR, and its conformational change is important for separation of the two associated JAK molecules and their subsequent activation^32^. The box 2 motif consisting of aromatic and acidic residues is less conserved and not required for JAK association but is important for kinase activation^33^.

The lamprey GHR has 6 cytoplasmic Tyr residues, and among them the Tyr^662^ and Tyr^693^ are aligned with the Tyr^595^ and Tyr^627^ of human GHR, respectively. In human GHR, several C-terminal Tyr residues are characterized as essential for the phosphorylation and activation of signal transducer and activator of transcription 5 (STAT5)^34,35^ and subsequently regulation of the expression of downstream genes, including IGF-1^36^. Among them, the Tyr^595^ and Tyr^627^ residues of human GHR are particularly important for STAT5 phosphorylation, and their mutation is associated with GHR-dependent impairment of STAT5 activation and severe short stature^37^. The lamprey PRLR has 5 cytoplasmic Tyr residues, and 2 C-terminal Try residues are aligned with the Tyr residues that are well conserved in gnathostome PRLRs and characterized as the primary sites for the phosphorylation and activation of STAT5^38^. It appears that the pairs of Tyr residues are conserved for the PRLRs, as well as for the GHRs, across both agnathans and gnathostomes, likely mediating intracellular signalling activation and transduction. Overall, the conservation of the functional motifs, including the extracellular motifs, box 1 and 2, and C-terminal Tyr residues, further supports our assignment of PRLR and GHR to the sea lamprey receptors. Moreover, these conserved motifs should enable the sea lamprey receptors to activate JAK-STAT cascades and consequently mediate various cellular actions.

The mRNAs encoded by the sea lamprey GHR and PRLR are expressed broadly among tissues similar to their teleost orthologs^10,39,40^. High levels of GHR mRNA were detected in the liver of sea lamprey, a pattern similar to the prominent hepatic expression of teleost GHR genes^10,11,39^. Sea lamprey GH has been shown to stimulate IGF expression in the lamprey liver^20^ as in teleost fish and other gnathostome species^10,11,36,39^. The sea lamprey GHR is likely the major receptor to bind GH in the liver, mediating GH action in stimulating IGF expression, due to its high abundance; its mRNA levels in the liver were about 25-time higher than those of PRLR (Fig. 3). Based on the analysis of the functional motifs in the sea lamprey GHR, the conserved cytoplasmic Tyr residues may be involved in STAT5 activation and subsequently regulation of IGF expression, as in human GHR^36^. The high transcription of lamprey GHR in the heart is similar to the pattern shown for a teleost GHR subtype^40,41^. The pattern of lamprey PRLR mRNA expression (high in the brain; moderate in the gill, kidney and gut) is opposite that of the pattern of PRLR mRNA expression in teleost (e.g., high in the osmoregulatory tissues, but less in the brain)^13,40^. Interestingly, shark PRLR is predominantly expressed in the pituitary gland with unknown function^5^ and mammalian PRLR has wide distribution in the central nervous system and appears important for regulating parental behavior as well as appetite and food intake^42^. Lamprey PRLR may mediate similar roles in the brain of lampreys.

The well-defined actions of GH in the regulation of growth and metabolism in gnathostomes^10^ are largely unknown in agnathans. In this study we focus on the sea lamprey metamorphosis, a particular life stage that involves reorganization of organs and remodelling of body to transform FW ammocoetes (larvae) to juveniles that can commence marine life. The ability to osmoregulate in SW is not present in ammocoetes but develops during metamorphosis^19^, and is similar to the parr-smolt transformation of salmonids^43^. Salinity tolerance is accompanied by increased Na^+^/K^+^-ATPase activity and the proliferation and differentiation of ionocytes in the lamprey gill^18,44^. In Atlantic salmon (*Salmon salar*), the acquisition of salinity tolerance is mediated by several hormones, and among them GH is essential for SW adaption. Both plasma GH levels and branchial GHR mRNA levels are upregulated in smoltification of salmon^45,46^. The observation of high branchial lamprey GHR mRNA levels coincident with developmental increases in osmoregulation indicates that GHR signalling likely plays a similar role in sea lamprey gill reorganization for salinity tolerance. Further upregulation of branchial GHR mRNA levels were seen in the post-metamorphic downstream migrants as well as in the SW-acclimated juveniles. The current observations indicate a potential role of the GHR in the acquisition of SW tolerance as seen in salmonids and other euryhaline teleost.

The lamprey PRLR mRNA levels were also upregulated in the gill during metamorphic stages, but earlier (by stage 5) than the increase in the GHR expression, and no further changes in the PRLR expression were observed in downstream migrants or in SW-acclimated juveniles. Moreover, downregulation of the PRLR mRNA levels was observed in the anterior intestine of SW-exposed juveniles compared to ammocoetes, whereas upregulation of GHR mRNA levels was observed in this tissue. These observations indicate divergent actions of the lamprey PRLR and GHR in the gill and anterior intestine during metamorphosis and SW acclimation.

In the liver, the lamprey GHR and PRLR mRNA levels were both reduced in the juveniles compared to ammocoetes; however, IGF mRNA levels displayed no significant change. There was no correlation of GHR or PRLR with IGF mRNA levels. GHR and PRLR have well-known roles in regulating intermediary metabolism in gnathostomes^10,11,47^. The importance of the changes in GHR and PRLR expression in the liver and the relative role(s) of GHR and PRLR in metabolism of lamprey remain to be investigated.

To date, only GH has been identified in lamprey. Due to the similarity in their structural motifs, both lamprey GHR and PRLR may be capable of binding GH. Such promiscuity already has been observed in the gnathostomes as GHRs and PRLRs can both bind GH^48,49^. Even with a single ligand, it is possible to have specific actions of GH in lamprey as a result of the differential distribution of GHR and PRLR (in both time and space), differential binding, and differential linkage of GHR and PRLR to activation of intracellular signalling systems. Because GH, PRL and SL likely arose in a vertebrate ancestor^26^, it is also possible that PRL has yet to be identified in lamprey genome assemblies due to the gaps in the current genome assemblies.

In conclusion, we have isolated and characterized two distinct genes encoding class-I cytokine receptors from the lampreys. The similarity of their gene structures, conservation of structural motifs, and the information from phylogenetic analysis indicate that the lamprey receptors are the orthologs of GHR and PRLR in gnathostomes. These findings suggest that distinct GHR and PRLR arose in the vertebrate lineage prior to the emergence of the lamprey branch of agnathans. This study also provides novel insight into the functional evolution GHR and PRLR in vertebrates. The distinct but overlapping pattern of expression of sea lamprey GHR and PRLR genes in tissues, as well as the observation that they are differentially expressed during metamorphosis and following SW exposure, support that the lamprey GHR and PRLR play different functional roles. The observed increase in branchial expression of lamprey GHR (but not of PRLR) following exposure to SW, an action that is conserved in euryhaline teleost, suggests that SW adaptation was one of the early functions of the GHR-GH system in vertebrates, while the mechanism underlying the participation of GHR-GH in lamprey gill reorganization for salinity tolerance requires further elucidation.

## Methods

### Experimental animals

Sea lampreys were collected between June and October via electrofishing or Fyke net capture from the Saw Mill River (Montague, MA, USA). Ammocoetes were captured in July, August, and October 2016; transformers at metamorphic stage 1 were captured in July; transformers at stages 5–6 were captured in August, and transformers in stage 7 were captured in October by electrofishing. Fully transformed downstream migrants were captured in November in the same river, approximately 220 km from the ocean.

Sea lampreys used in the metamorphosis analysis were euthanized and sampled immediately upon capture in the field; animals used for tissue distribution and salinity acclimation experiments were transferred back to the laboratory (USGS Conte Anadromous Fish Research Laboratory, Turners Falls, MA, USA) and maintained in 1-m diameter tanks supplied with 4 L min^−1^ Connecticut River water. Juveniles for tissue distribution analysis were sampled from these tanks, including three from fresh water (FW) and three from seawater (SW) [body mass (BM) 3–7 g and body length (BL) 14–17.7 cm]. For comparison of salinity effects on juveniles, FW juveniles (BM 3.5–5.2 g and BL13.7–16 cm) were placed into 15 L recirculating glass aquaria at 15 °C under a simulated natural photoperiod. Salinity (35 ppt, SW) was prepared from artificial sea salt (Crystal Sea Salt, Baltimore, MD, USA), and animals (BM 3.3–6.1 g and BL 13.9–16.2 cm) were acclimated to SW for 3 weeks before sampling. Ammocoetes (BM 1–4 g and BL 9–15 cm) in FW were included for comparison, but they were not subjected to SW exposure because they were intolerant to salinity greater than 8 ppt.

During sampling in the field and laboratory, animals were euthanized using a lethal dose of MS-222 (400 mg L^−1^ buffered with NaHCO_3_, pH 7.0) (Argent Chemical Laboratories, Redmond, WA, USA) and sampled for tissues, which were immediately frozen by liquid nitrogen and stored at -80 °C. All procedures involving animals were conducted in accordance with the Guide for Care and Use of Laboratory Animals (National Research Council, Washington, DC) and approved by the USGS Leetown Science Center Institutional Animal Care and Use Committee.

### Database searches and gene prediction

The amino acid sequences of truncated GHR/PRLR isoform of sea lamprey (Genbank no. AGN52917.1), GHR (XP015223497) and PRLR (XP006627271) from spotted gar were used to query the sea lamprey genome in the Ensembl genome browser (http://www.ensembl.org), Arctic lamprey (*Lethenteron camtschaticum*) genome^22^ LetJap1.0, and sea lamprey germline genome^21^ Pmar_germline 1.0 from the National Center for Biotechnology Information (NCBI) assembly resource (https://www.ncbi.nlm.nih.gov/assembly) with the TBLASTN algorithm. Gene sequences were predicted by Augustus gene prediction server (http://bioinf.uni-greifswald.de/augustus/). Specific primers for PCR were designed based on the predicted gene sequences.

The sea lamprey PRLR sequence was used to query Pmar_germline 1.0 with TBLASTN. Among the identified sequences producing significant alignment, a sequence that did not belong to the sea lamprey PRLR but is within an unknown protein that was predicted by Augustus prediction server. Alignment of the predicted protein with the sea lamprey PRLR and gnathostome GHRs and PRLRs showed several conserved motifs. Thus, this protein was subjected to gene cloning and sequencing. This was revealed as the sea lamprey putative GHR. The gene sequences of sea lamprey GHR and PRLR were queried with TBLASTN against the Arctic lamprey genome^22^ LetJap1.0 and the inshore hagfish genome^23^ assemblies, to search for the gene sequences of PRLR and GHR from Arctic lamprey and inshore hagfish GHR/PRLR-like. The exons and splicing junctions were defined using the Ensembl protein report service.

### Cloning and sequencing of the sea lamprey genes

The sea lamprey PRLR gene was amplified by RT-PCR using Platinum *Taq* DNA polymerase high-fidelity PCR reagents (Invitrogen, Carlsbad, CA, USA). The sea lamprey GHR was amplified by RT-PCR using Platinum II *Taq* DNA Polymerase with high GC-rich PCR performance (Invitrogen, Vilnius, Lithuania). PCR products were cloned to pGEM-T vector (Promega Corp, Madison, WI, USA) and subcloned in chemically competent *E*. *coli*. Plasmids from at least three positive colonies were extracted and sequenced by Macrogen USA (Rockville, MD, USA). The primer information is listed in Supplemental Table S1.

### RNA extraction and cDNA synthesis

Total RNAs were isolated with the RNeasy Mini kit (Qiagen, Hilden, Germany) or with TRIzol reagent (Invitrogen). cDNAs were synthesized in a 20-µl reaction with 5 μg total RNA template using the SuperScript III First-Strand Synthesis System (Invitrogen, Carlsbad, CA, USA) or with 2 μg RNA using the High-Capacity cDNA Reverse Transcription kit (Applied Biosystems, Vilnius, Lithuania). Because of low RNA yields, we used 200 ng RNA from pituitary gland and 1 µg from brain.

### Real-time PCR

Quantitative real-time PCR (qPCR) was used to quantify steady-state mRNA levels; 20–37.5 ng cDNA was used as templates for target genes (GHR, PRLR and IGF) or 6.25 ng cDNA for reference genes [β-ACTIN, glyceraldehyde 3-phosphate dehydrogenase (GAPDH) and elongation factor a (EF1A)]. The qPCR reaction included 300 nM primers, the cDNA templates, and *Power* SYBR green PCR master mix (Applied Biosystems, Warrington, UK) in 20 µl of final volume. The primers are listed in Supplemental Table S1. No PCR product was amplified from the negative control, which was prepared with RNA and the cDNA reserves transcription reagents without reverse transcriptase. Data from the qPCR runs were collected with ABI Prism 7300 sequence detection system (Applied Biosystems). The tested gene copy numbers were normalized by EF1A, GAPDH, or β-ACTIN, depending on which gene has stable transcription in the experimental groups. cDNA standards were prepared with the linearized plasmids (the pGEM-T vector constructed with insert gene) to make the standard curves. PCR efficiencies in the qPCR with the cDNA standards were between 95% and 105%. The cDNA copy numbers were calculated from the standards curves.

### Sequence alignment and phylogenetic analysis

Alignment of amino acid sequences was made using the MUSCLE algorithm applied through AliView software with default settings^50^. The sequence similarities were calculated by Clustal Omega, an online alignment tool provided by EMBL-EBI (https://www.ebi.ac.uk/services). Phylogenetic analysis was carried out using the likelihood-based Phylogenetic Maximus Likelihood method (PhyML). PhyML trees were made using the PhyML 3.1 algorithm through Seaview 4.7^51^ with the following settings: LG model of amino acid substitution and approximate Likelihood Ratio Tests (aLRT) with SH-like supports were selected; amino acid equilibrium frequencies were estimated from the alignments (empirical); the proportion of invariable sites was optimized; the number of substitution rate categories was increased from 4 to 8; the starting tree was estimated using BioNJ with optimized tree topology; both NNI and SPR tree improvement methods were considered to estimate the best tree topology^6^. Branch lengths of the tree are proportional to the number of substitutions per site.

The selection of gnathostome representatives for the phylogenetic analysis was based on the GHR and PRLR gene trees in Ensembl, including the ray-finned fishes (spotted gar and teleost fishes) and the lobe-finned fishes [coelacanth (*Latimeria chalumnae*) and tetrapods]. Spotted gar sequences were used as a relative dating point for the fish-specific genome duplication. The selection of representative teleost was based on the availability of more complete genome datasets, including zebrafish (*Danio rerio*), Japanese medaka (*Oryzias latipes*), tetraodon (*Tetraodon nigroviridis*), fugu (*Takifugu rubripes*), Nile tilapia (*Oreochromis niloticus*), platy fish (*Xiphophorus maculatus*), tiger tail seahorse (*Hippocampus comes*), barramundi perch (*Lates calcarifer*), and three-spine stickleback (*Gasterosteus aculeatus*). The salmonids that have gone through the salmonid-specific tetraploidization were also included, e.g., Atlantic salmon (*Salmo salar*), rainbow trout (*Oncorhynchus mykiss*) and huchen (*Hucho hucho*). For simplicity of the figure, elephant shark (*Callorhinchus milii*) and brownbanded bamboo shark (*Chiloscyllium punctatum*) were selected as the representatives of cartilaginous fishes. The accession numbers of the sequences of GHR, PRLR and CRFA4 are listed in Supplemental Table S2. A duplicated ligand-binding domain in chicken and lizard PRLRs was removed from the phylogenetic analysis. The partial sequence of coelacanth PRLR was excluded from phylogenetic analysis to avoid extra alignment gap and to improve aLRT support. The selection of CRFA4 sequences for phylogenetic analysis was according to the corresponding gene tree (No. ENSGT00940000154851) in the Ensembl. Only substantially long sequences that contain the three essential parts of the receptor were selected for phylogenetic analysis. The CRFA4 sequences have been only found in coelacanth, spotted gar, and teleost fish, but not in cartilaginous fish and tetrapods^6^.

### Statistical analysis

qPCR data were analyzed by one-way ANOVA followed by a Turkey post hoc test (all pairwise comparisons) or Dunnett post-test (compare all treatments to control) conducted in software GraphPad Prism (La Jolla, CA, USA) to identify differences among groups; differences were considered significant at P < 0.05.

## Supplementary information

Supplemental tables and figures.
