# Supplementary material for: Divergent genes encoding the putative receptors for growth hormone and prolactin in sea lamprey display distinct patterns of expression
Source: Sci Rep. 2020 Feb 3;10:1674. doi: 10.1038/s41598-020-58344-5 (PMC6997183; doi:10.1038/s41598-020-58344-5)
Supplement: Supplementary file 1 — Supplemental tables and figures. [file 41598_2020_58344_MOESM1_ESM.doc]

Supplemental tables and figures for “**Divergent genes encoding the putative receptors for growth hormone and prolactin in sea lamprey display distinct patterns of expression**” by Gong N, Ferreira-Martins D, McCormick SD and Sheridan MA.

**Supplemental table S1:** Primer used for RT-PCR and real-time qPCR of the genes from sea lamprey.

| Primers and gene sequences | Application |
| --- | --- |
| Forward: CAGATCCGGTGGTTCTGGTC | RT-PCR of intracellular part of PRLR |
| Reverse: GGTTCTTCCGGTGAACCCTT |
| Forward: AGCCGAGGACGGCATGCGAG | RT-PCR of full-length PRLR |
| Reverse: TCACTTTCCCCCCCCACTCCC |
| Forward: ATGTGCGGGAGATGTGG | RT-PCR of 5’ part of GHR |
| Reverse: GCCACAACTGTCGGTCAAGA |
| Forward: CGGTGGAGCGGTGTCAATC | RT-PCR of middle part of GHR |
| Reverse: GCCCTTGACGACGTCCTTG |
| Forward: CTCCTCGCCACGCTACTG | RT-PCR of 3’ part of GHR |
| Reverse: TGAATGCAAGCGCAATGGAC |
| Forwards: GGCATAAACCAAGTGGCGCA | qPCR of PRLR |
| Reverse: CGATCAGGGTGACAAGGAACA |
| Forwards: CTCCTCGCCACGCTACTG | qPCR of GHR |
| Reverse: TCATGCGGTTGAACTCCTCC |
| Forwards: GCTCGTCGATGCCCTCC | qPCR of IGF |
| Reverse: CACGATGCCCTTCTTGGGT |
| Forwards: CCTTCCTCGGAATGGAGTCG | qPCR of β-ACTIN |
| Reverse: GTACAGGTCCTTGCGGATGT |
| Forwards: ATTCGTCCAGCTCCGTTCAG | qPCR of GAPDH |
| Reverse: ATGTCTTCCTTTTGCACGGC |
| Forwards: GAAGCCAGCACCAACATGC | qPCR of EF1A |
| Reverse: GATGGCATCCAGAGCCTCC |

**Supplemental table S2: GenBank and Ensembl accession numbers of GHRs, PRLRs, and CRFA4s used for phylogenetic analysis.**

| Species | Gene number | Species | Gene number |
| --- | --- | --- | --- |
| Sea lamprey (*Petromyzon marinus*) | GHR, MK593139 | Human | PRLR, M31661.1 |
| Sea lamprey | PRLR, MF685336 | Rat | PRLR, M57668.1 |
| Human (*Homo sapiens* | GHR, NP_001229335.1 | Mouse | PRLR, L14811.1 |
| Rat (*Rattus norvegicus*) | GHR, NM_017094.1 | Opossum | PRLR, XP_016287101 |
| Mouse (*Mus musculus*) | GHR, NP_001273299.1 | Chinese soft-shelled turtle | PRLR, XM_014570234.1 |
| Opossum (*Monodelphis domestica* | GHR, NM_001032976.1 | Chicken | PRLR, NM_204854.1 |
| Chinese soft-shelled turtle (*Pelodiscus sinensis* | GHR, XP_006139558.1 | Coelacanth | PRLR, ENSLACG00000001448 |
| Chicken (*Gallus gallus*) | GHR, NM_001001293.1 | Frog | PRLR, AB030443.1 |
| Frog (*Xenopus laevis*) | GHR, ENSXETG00000005560 | Spotted gar (*Lepisosteus oculatus*) | PRLR, ENSLOCP00000014539 |
| Coelacanth (*Latimeria chalumnae*) | GHR, ENSLACT00000006305.1 | Zebrafish | PRLRa, ENSDART00000128859 |
| Elephant shark (*Callorhinchus milii*) | GHR, XM_007901587.1 | Rainbow trout | PRLRa, AF229197.1 |
| Bamboo shark (*Chiloscyllium punctatum)* | GHR, Chipu0013466.t1 | Atlantic salmon | PRLRa, XM_014138867.1 |
| Cloudy catshark (*Scyliorhinus torazame)* | GHR, Scyto0020787.t1 | Fugu | PRLRa, ENSTRUT00000029978.1 |
| Zebrafish (*Danio rerio*) | GHRa, ENSDARG00000054771 | Medaka | PRLRa, ENSORLT00000007444.1 |
| Platy fish (*Xiphophorus maculatus*) | GHRa, ENSXMAG00000015913 | Tilapia | PRLRa, NM_001279548.1 |
| Medaka (*Oryzias latipes*) | GHRa, ENSORLT00000005070.1 | Platy fish | PRLRa, XP_005798743 |
| Tilapia (*Oreochromis niloticus*) | GHRa, AY973232.1 | Rainbow trout | PRLRb, NC_035082.1 |
| Fugu (*Takifugu rubripes*) | GHRa, ENSTRUT00000038560 | Atlantic salmon | PRLRb, NC_027323.1 |
| Rainbow trout (*Oncorhynchus mykiss*) | GHRa1, XP_021462199.1 | Fugu | PRLRb, ENSTRUT00000043279.1 |
| Atlantic salmon (*Salmo salar*) | GHRa1, XM_014171137.1 | Platy fish | PRLRb, XP_023199074.1 |
| Rainbow trout | GHRa2, JQ408978.2 | Medaka | PRLRb, ENSORLT00000000408.1 |
| Zebrafish | GHRb, ENSDART00000163003 | Tilapia | PRLRb, ENSONIT00000021367.1 |
| Platy fish | GHRb, ENSXMAG00000016511 | Zebrafish | PRLRb, ENSDART00000067541 |
| Medaka | GHRb, ENSORLT00000018552.1) | Coelacanth | CRFA4, ENSLACT00000005176.1 |
| Fugu | GHRb, ENSTRUT00000033116 | Spotted gar | CRFA4, ENSLOCT00000011850.1 |
| Tilapia | GHRb, AY973233.1 | Cave fish | CRFA4, ENSAMXT00000016499.2 |
| Rainbow trout | GHRb1, NP_001118007.1 | Channel catfish | ENSIPUT00000017651.1 |
| Atlantic salmon | GHRb1, NP_001117048.1 | Cod | CRFA4, ENSGMOT00000009386.1 |
| Rainbow trout | GHRb2, AY573600.2 | Spotted green pufferfish | CRFA4, AY374476.1 |
| Atlantic salmon | GHRb2, NP_001117066.1 | Tetraodon | CRFA4, ENSTNIT00000004284.1 |
| Fugu | CRFA4, ENSTRUT00000029901.2 | Tilapia | CRFA4, ENSONIT00000004597.1 |
| Japanese medaka | CRFA4, ENSORLT00020008775.1 | Platy fish | CRFA4, ENSXMAT00000037438.1 |


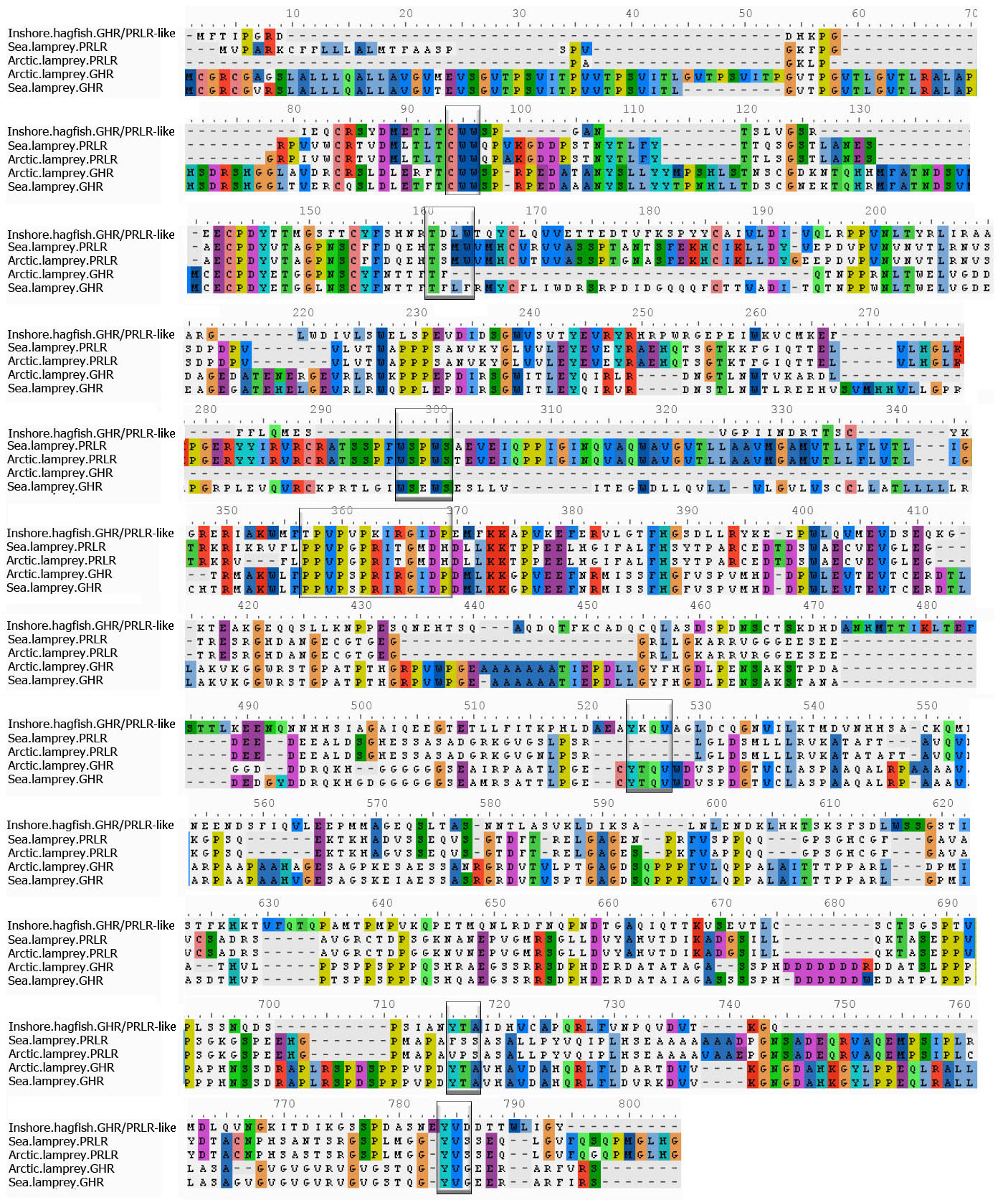


**Supplemental figure S1:** Amino acid alignment of the PRLRs and GHRs from sea lamprey and Arctic lamprey and the GHR/PRLR-like from inshore hagfish, using the MUSCLE algorithm. The conserved motifs and intracellular tyrosine residues are framed. The sequences of Arctic lamprey GHR and hagfish GHR/PRLR-like are incomplete, due to the gaps within the genome scaffolds.

**
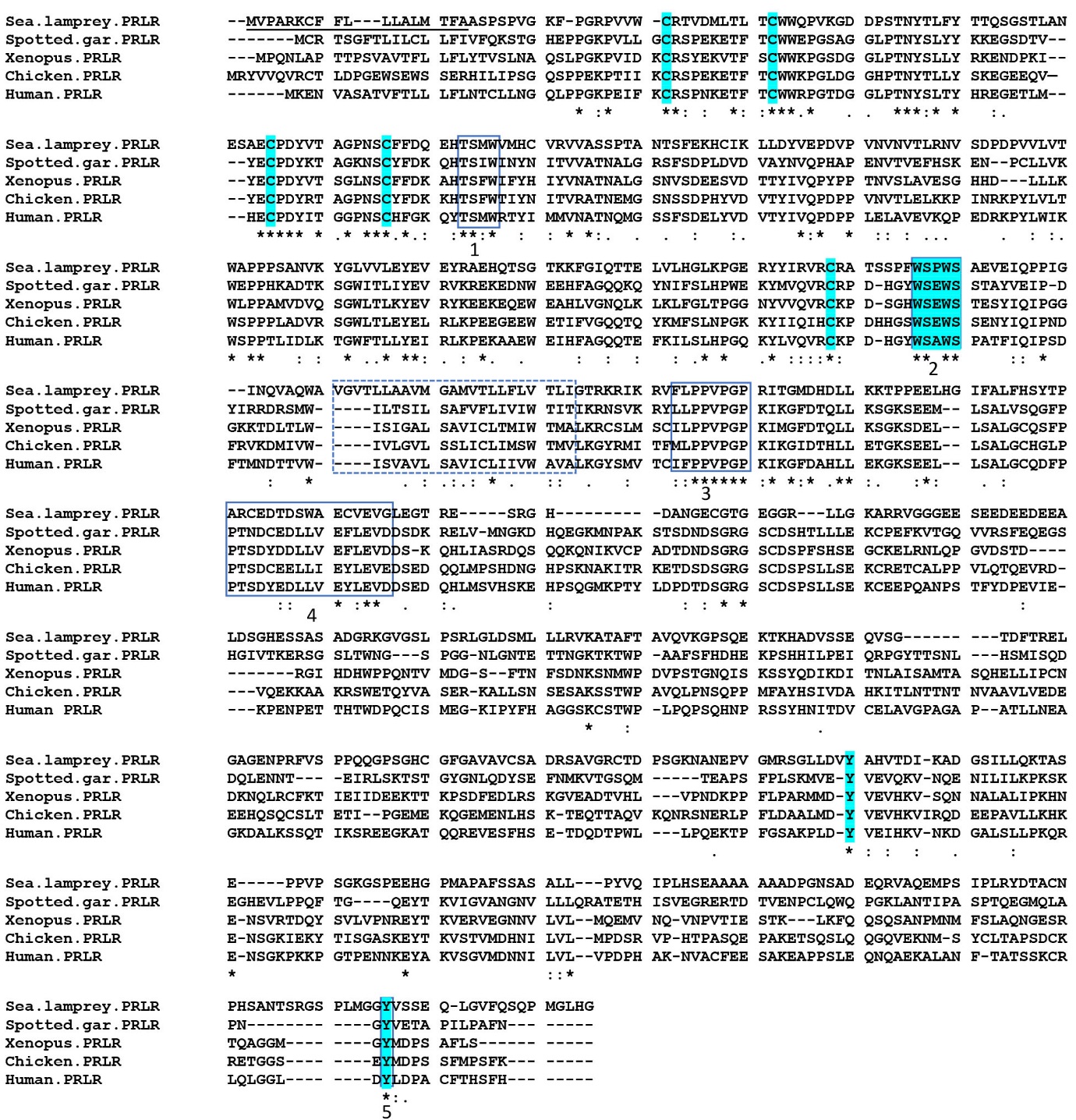
Supplemental figure S2:** Amino acid alignment of sea lamprey PRLR with PRLRs from human, chicken, frog (*Xenopus tropicalis)* and spotted gar. The duplicated ligand-binding domain of chicken PRLR sequence was removed from amino acid alignment. The conserved motifs are framed and numbered, and the conserved cystine and tyrosine resides are highlighted. The predicted signal peptide sequence is underlined, and the predicted transmembrane domain is in dashed box. Asterisks indicate amino acids that are conserved in all sequences, whereas colons and dots indicate decreasing levels of similarity.

**
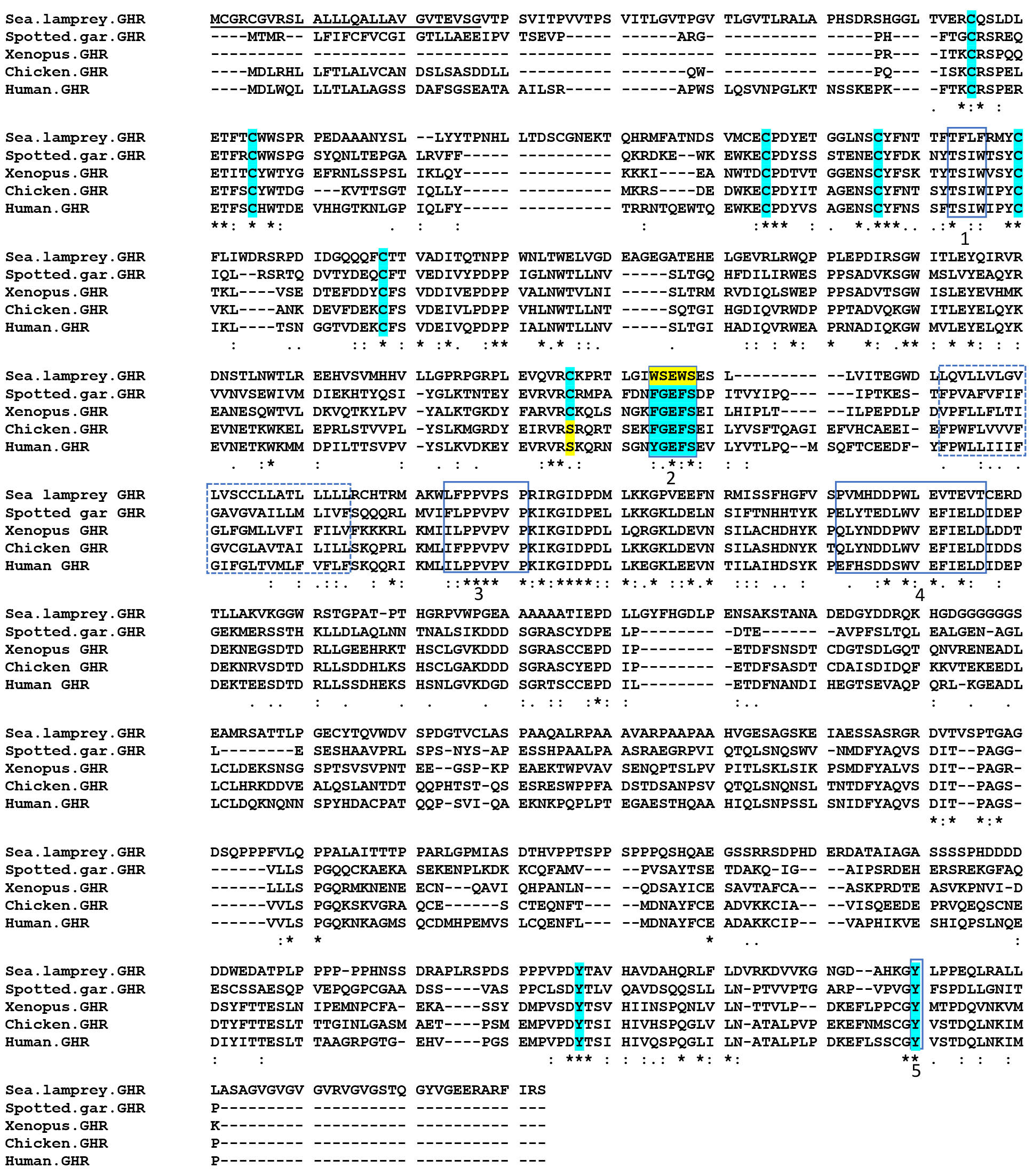
Supplemental figure S3:** Amino acid alignment of sea lamprey GHR with GHRs from human, chicken, frog (*Xenopus tropicalis)*, and spotted gar. The conserved motifs are framed and numbered, and the conserved cystine and tyrosine resides are highlighted. The predicted signal peptide sequence is underlined, and the predicted transmembrane domain is in dashed box. Asterisks indicate amino acids that are conserved in all sequences, whereas colons and dots indicate decreasing levels of similarity.
